# Draft Genome Sequence of *Talaromyces adpressus*

**DOI:** 10.1128/genomeA.01430-17

**Published:** 2018-01-11

**Authors:** Gabriella Cerullo, Jos Houbraken, Zoraide Granchi, Olimpia Pepe, Simona Varriale, Valeria Ventorino, Thomas Chin-A-Woeng, Martin Meijer, Ronald P. de Vries, Vincenza Faraco

**Affiliations:** a Department of Chemical Sciences, University of Naples Federico II, Complesso Universitario Monte S. Angelo, Naples, Italy; b Department of Applied and Industrial Mycology, Westerdijk Fungal Biodiversity Institute, Utrecht, The Netherlands; c GenomeScan B.V., Leiden, The Netherlands; d Department of Agricultural Sciences, University of Naples Federico II, Portici (NA), Italy; e Fungal Physiology, Westerdijk Fungal Biodiversity Institute & Fungal Molecular Physiology, Utrecht University, Utrecht, The Netherlands

## Abstract

Here we present the draft genome sequence of the fungus *Talaromyces adpressus* A-T1C-84X (=CBS 142503). This strain was isolated from lignocellulosic biomass of *Arundo donax* during biodegradation under natural conditions in the Gussone Park of the Royal Palace of Portici, Naples, Italy.

## GENOME ANNOUNCEMENT

*Talaromyces adpressus*, a recently described species belonging to section *Talaromyces*, was until now only known from the indoor environment in China (1). The strain sequenced here, CBS 142503, belonging to the microbial collection (strain number A-T1C-84X) of the Division of Microbiology, Department of Agricultural Sciences of the University of Naples Federico II, was isolated from lignocellulosic biomass of *Arundo donax* during biodegradation under natural conditions in the Gussone Park of the Royal Palace of Portici, Naples, Italy.

This strain was selected for its abilities to synthetize different enzymes having potentially synergistic actions on lignocellulose conversion, such as endo- and exocellulase, cellobiase, xylanase, pectinase, and laccase, assayed using specific solid media as previously described by Ventorino et al. (2), and to produce a feruloyl esterase (FAE) active against *p*-nitrophenyl-ferulate.

*Talaromyces adpressus* CBS 142503 was cultivated in complete medium (3). Mycelium was sampled after 48 h of growth, and genomic DNA was extracted using a cetyltrimethylammonium bromide (CTAB)-based extraction buffer (4). Concentration and quality of the samples were determined using the Life Technology Qubit and 0.6% agarose gel, respectively. Genome sequencing was performed at GenomeScan. The NEBNext Ultra DNA library preparation kit from Illumina (catalog number NEB E7370S/L) was used according to the manual for library preparation. Quality and yield after sample preparation were measured with Bioanalyzer (Agilent Technologies).

Clustering and DNA sequencing using the Illumina cBot and HiSeq 2500 were performed according to the manufacturer’s protocols using a concentration of 8.0 pM DNA, standard Illumina primers, and HiSeq control software HCS v2.2.58. Image analysis, base calling, and quality check were performed with the Illumina data analysis pipeline RTA v1.18.64 and Bcl2fastq v1.8.4. Reads were trimmed for adapter sequences and filtered for sequence quality using the in-house tool FASTQFilter v2.05. The short-read genome assembler Abyss v1.3.7 (5) was used for assembly. An optimization for k-mer length was performed as in previous fungal genome assemblies. A length of 64 bp was found to give the best results, optimized for the smallest number of scaffolds with longer average length. Scaffolds shorter than 500 bp, unlikely to contain complete coding sequences, were removed. The 36.12-Mb genome resulted from the assembly of 599 contigs. GC content was of 46.22% as assessed by QUAST (6).

### Accession number(s).

The draft genome sequence of *T. adpressus* A-T1C-84X (=CBS 142503) has been deposited at DDBJ/ENA/GenBank under accession number NHZS00000000. The version described in this paper is version NHZS01000000. The BioProject in GenBank is PRJNA381192. The strain is available from the CBS culture collection (http://www.westerdijkinstitute.nl) housed at the Westerdijk Institute (Utrecht, The Netherlands).

## References

[1] Chen AJ, Sun BD, Houbraken J, Frisvad JC, Yilmaz N, Zhou YG, Samson RA 2016 New *Talaromyces* species from indoor environments in China. Stud Mycol 84:119–144. doi:10.1016/j.simyco.2016.11.003. 28070136 PMC5219591

[2] Ventorino V, Aliberti A, Faraco V, Robertiello A, Giacobbe S, Ercolini D, Amore A, Fagnano M, Pepe O 2015 Exploring the microbiota dynamics related to vegetable biomasses degradation and study of lignocellulose-degrading bacteria for industrial biotechnological application. Sci Rep 5:8161. doi:10.1038/srep08161. 25641069 PMC4648445

[3] de Vries RP, Burgers K, van de Vondervoort PJI, Frisvad JC, Samson RA, Visser J 2004 A new black *Aspergillus* species, *A*. *vadensis*, is a promising host for homologous and heterologous protein production. Appl Environ Microbiol 70:3954–3959. doi:10.1128/AEM.70.7.3954-3959.2004. 15240269 PMC444756

[4] Hildén K, Martinez AT, Hatakka A, Lundell T 2005 The two manganese peroxidases Pr-MnP2 and Pr-MnP3 of *Phlebia radiata*, a lignin-degrading basidiomycete, are phylogenetically and structurally divergent. Fungal Genet Biol 42:403–419. doi:10.1016/j.fgb.2005.01.008. 15809005

[5] Simpson JT, Wong K, Jackman SD, Schein JE, Jones SJM, Birol I 2009 ABySS: a parallel assembler for short read sequence data. Genome Res 19:1117–1123. doi:10.1101/gr.089532.108. 19251739 PMC2694472

[6] Gurevich A, Saveliev V, Vyahhi N, Tesler G 2013 QUAST: quality assessment tool for genome assemblies. Bioinformatics 29:1072–1075. doi:10.1093/bioinformatics/btt086. 23422339 PMC3624806

